# Bioinformatics resource manager v2.3: an integrated software environment for systems biology with microRNA and cross-species analysis tools

**DOI:** 10.1186/1471-2105-13-311

**Published:** 2012-11-23

**Authors:** Susan C Tilton, Tamara L Tal, Sheena M Scroggins, Jill A Franzosa, Elena S Peterson, Robert L Tanguay, Katrina M Waters

**Affiliations:** 1 Computational Biology and Bioinformatics, Pacific Northwest National Laboratory, Richland, WA, USA; 2 Department of Environmental and Molecular Toxicology, Oregon State University, Corvallis, OR, USA; 3 Scientific Data Management, Pacific Northwest National Laboratory, Richland, WA, USA; 4 Current address: Integrated Systems Toxicology Division, National Health and Environmental Effects Research Laboratory, U.S. Environmental Protection Agency, Research Triangle Park, NC, USA

**Keywords:** Systems biology, Genomics, MicroRNA, Bioinformatics, Zebrafish

## Abstract

**Background:**

MicroRNAs (miRNAs) are noncoding RNAs that direct post-transcriptional regulation of protein coding genes. Recent studies have shown miRNAs are important for controlling many biological processes, including nervous system development, and are highly conserved across species. Given their importance, computational tools are necessary for analysis, interpretation and integration of high-throughput (HTP) miRNA data in an increasing number of model species. The Bioinformatics Resource Manager (BRM) v2.3 is a software environment for data management, mining, integration and functional annotation of HTP biological data. In this study, we report recent updates to BRM for miRNA data analysis and cross-species comparisons across datasets.

**Results:**

BRM v2.3 has the capability to query predicted miRNA targets from multiple databases, retrieve potential regulatory miRNAs for known genes, integrate experimentally derived miRNA and mRNA datasets, perform ortholog mapping across species, and retrieve annotation and cross-reference identifiers for an expanded number of species. Here we use BRM to show that developmental exposure of zebrafish to 30 uM nicotine from 6–48 hours post fertilization (hpf) results in behavioral hyperactivity in larval zebrafish and alteration of putative miRNA gene targets in whole embryos at developmental stages that encompass early neurogenesis. We show typical workflows for using BRM to integrate experimental zebrafish miRNA and mRNA microarray datasets with example retrievals for zebrafish, including pathway annotation and mapping to human ortholog. Functional analysis of differentially regulated (p<0.05) gene targets in BRM indicates that nicotine exposure disrupts genes involved in neurogenesis, possibly through misregulation of nicotine-sensitive miRNAs.

**Conclusions:**

BRM provides the ability to mine complex data for identification of candidate miRNAs or pathways that drive phenotypic outcome and, therefore, is a useful hypothesis generation tool for systems biology. The miRNA workflow in BRM allows for efficient processing of multiple miRNA and mRNA datasets in a single software environment with the added capability to interact with public data sources and visual analytic tools for HTP data analysis at a systems level. BRM is developed using Java™ and other open-source technologies for free distribution (http://www.sysbio.org/dataresources/brm.stm).

## Background

MicroRNAs (miRNAs) are small ~22 nucleotide non-coding RNAs that function as post-transcriptional regulators of gene expression. Since miRNAs typically act on mRNA targets through sequence complementarity in the 3’UTR, it is possible to computationally predict miRNA target genes. Over 30% of human protein coding genes are predicted to be regulated by miRNAs, which are also highly conserved across species and associated with diverse biological processes and diseases, including nervous system development, cardiac function, metabolism and cancer [1-3]. miRNAs also demonstrate cross-talk with epigenetics in that they are subject to epigenetic regulation by methylation and are involved in establishing methylation patterns [4-6]. Given the importance of miRNAs in controlling gene regulation, it is necessary to develop computational tools to understand the biological and functional consequences of miRNAs in a growing number of model organisms.

There are several public resources available for miRNA research [3], including miRNA databases (miRBase; [7]) and target prediction algorithms such as TargetScan [2], PicTar [8] and miRanda [7]. Computational tools have been developed to query multiple prediction databases simultaneously (miRGen [9]) or incorporate experimentally validated targets (TarBase; [10]). miRGator [11] facilitates functional understanding of miRNAs by providing disease associations and gene set analysis for a reference database of curated miRNA and gene expression datasets. Other tools further the integration of experimentally derived human miRNA and mRNA datasets (MMIA [12], MAGIA [13]). However, no tools offer biologists the ability to integrate heterogeneous miRNA and mRNA datasets for multiple species within a platform that also provides additional functional analysis and visualization tools. Furthermore, computational tools for non-human research models, such as zebrafish, are in high demand as genome sequencing improves for these species.

The Bioinformatics Resource Manager (BRM) is a software environment developed for data retrieval, management, analysis and visualization of high-throughput (HTP) biological data [14]. BRM provides computational tools for biologists to merge datasets, cross-reference gene identifiers and add functional annotation from NCBI [15], UniProt [16], CMR [17], GO [18] or KEGG [19] databases. These tools simplify mundane analysis tasks of data reformatting and table merging that can be intensive, thereby providing a platform for straightforward integration of heterogeneous HTP datasets [20]. BRM further facilitates connectivity between data sources and visual analytic tools for more effective data mining within a single software environment. By utilizing the GAGGLE [21] communication framework, BRM can broadcast data to publically available tools, such as R, Cytoscape [22], TMeV [23] and DAVID [24], and then back to the BRM project browser for seamless data analysis [25]. In this way, BRM provides essential data management capabilities such that all data files and metadata associated with those files are permanently stored to the user profile.

We report here several new features in BRM v2.3 that provide miRNA resources and cross-species identifier retrieval for *Homo sapiens* (human), *Mus musculus* (mouse), *Rattus norvegicus* (rat), *Danio rerio* (zebrafish), and *Macaca mulatta* (macaque). The miRNA workflows within BRM allow for identification of conserved miRNA gene targets from multiple public databases or of potential regulatory miRNAs for genes of interest. BRM makes it easy to merge predicted target lists with experimental mRNA lists and analyze the resulting genes using clustering, functional enrichment and network modeling tools. It is now also possible to compare data across different species in BRM using the Ensembl ortholog database and to cross-reference or annotate identifiers for numerous species, including those listed above. These new features make BRM a convenient software program for both cross-species data analysis and determining the functional consequences of genes from HTP microarray or RNAseq platforms that are regulated by miRNAs.

## Implementation

BRM is implemented in Java and is a client–server based system using Enterprise Java Beans 2.0 technology. The server is also Java based and uses the JBOSS application server. A PostgreSQL database supports all data storage for the system. The client application is lightweight in that all data manipulation, query, and storage are on the server side.

In earlier versions of BRM, the data from public sources, such as NCBI and KEGG, were accessed on the fly with direct connections through various API’s (Shah et al., [2007]). However, due to restrictive licensing and a lack of API support by many data source providers, BRM changed its model to store a copy in its own server and update on a regular basis. Every six months the BRM team updates its data store to retrieve any updates from the many data sources it makes available. This has the added benefit of upgrading performance especially if the client and server are on the same network. It also allows for a complete stand-alone (no internet required) implementation if warranted.

BRM was designed to keep data transfer and client–server communication at a minimum to support performance and the user experience. That model still holds even while data volume has grown exponentially. Even so, there are limitations placed on certain types of queries that would overload the system. The users are informed a priori of long running queries and, at times, are only provided with a subset of results (up to 500k) at a time.

BRM v2.3 has also changed its deployment model to take advantage of Java Webstart technology which allows for clients to be automatically updated when needed. Each time a client application is started, the server is pinged for any updates in the code. Updates are then downloaded immediately behind the scenes. This model allows for more frequent bug-fixes and updates without user intervention to uninstall and reinstall for every update.

## Results and discussion

BRM v2.3 efficiently processes HTP datasets (e.g. microarray, RNAseq, proteomic) with the added capability to interact with public data sources and visual analytic tools. The addition of miRNA data sources and cross-species identifier retrieval to BRM creates a unique platform in which miRNA target prediction, merging with experimental mRNA data and downstream functional analysis and interpretation are possible within the same environment. This is particularly novel for non-human vertebrate species, such as zebrafish, which are not supported in other programs. We show here a typical workflow in BRM for integration of experimental zebrafish miRNA and mRNA microarray datasets with example retrievals for zebrafish, including pathway annotation and mapping to human orthologs.

### BRM overview

**Figure 1:**
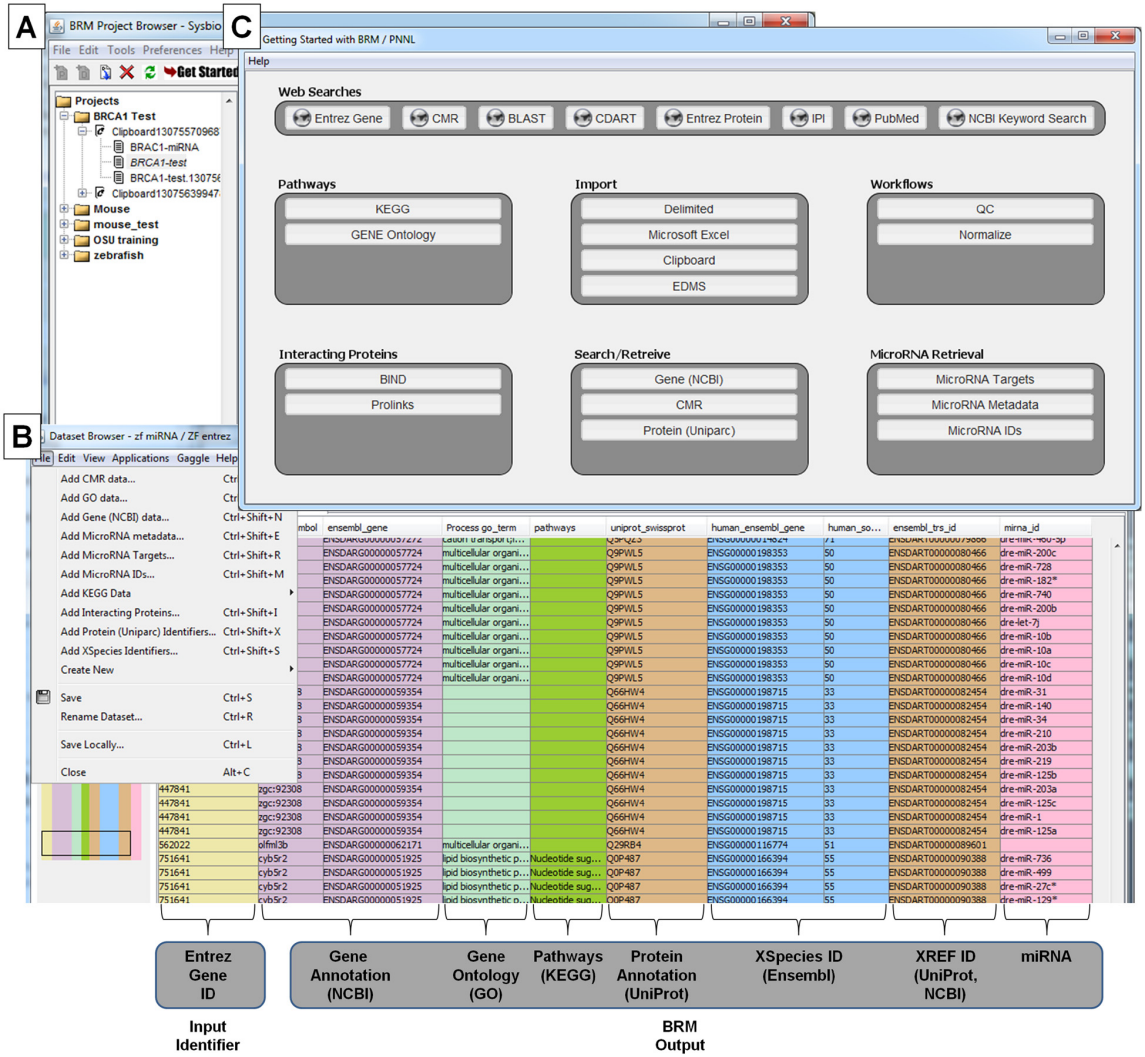
**The Bioinformatics Resource Manager Client: Example retrievals for zebrafish.** BRM features three primary interfaces including (**A**) the Project file browser, (**B**) Dataset table browser and (**C**) the Get Started menu for accessing tools and datasets. The Get Started menu provides access to most data import and retrieval options. Other retrieval options and analysis tools can also be accessed through the Dataset browser. Example retrievals for zebrafish annotation, cross-species identifiers and cross-reference identifiers are provided from NCBI, GO, KEGG, UniProt, Ensembl and miRNA databases in BRM.

As summarized in Table 1, BRM allows biologists to manage, process, analyze and visualize HTP data and also perform retrievals of batch annotations, cross reference identifiers and miRNA data necessary for systems biology research. BRM utilizes a familiar, easy to use, spreadsheet format with three primary interfaces that include the Project file browser, Dataset table browser and Get Started menu (Figure 1A-C, respectively). The Get Started menu provides access to most data import and retrieval options. Data can be imported in delimited or Excel file format or through pasting into a clipboard. The cross-species identifier query for ortholog mapping and miRNA retrieval options, including miRNA targets, miRNA metadata and miRNA IDs, are accessible through the Get Started menu or from within the dataset browser File menu (Figure 1B-C). Each of these retrievals, which are described below in more detail, are available for *Homo sapiens* (human), *Mus musculus* (mouse), *Rattus norvegicus* (rat), *Danio rerio* (zebrafish), and *Macaca mulatta* (macaque).

**Table 1:** Bioinformatics Resource Manager (BRM) v2.3 capabilities

**Data Management**		**XREF Identifier Retrieval**	
· Organization and Storage		· Gene Identifiers (NCBI)	
· Versioning		· Protein Identifiers (UniParc)	
· Meta-data tracking		· Cross Species Orthologs (Ensembl)	
· Importing data from clipboard, EDMS		· Prokaryotic Identifiers (CMR)	
**Data Processing and Editing**		**miRNA Datasource Retrieval**	
· Dataset Merging		· miRNA Predicted Targets	
· Table Features (OpenOffice Calc)		· (TargetScan, microCosm, microRNA)	
· Extraction of Embedded Data/IDs		· miRNA Identifiers	
· Column Merging/Splitting		· miRNA Metadata	
· Duplicate Row Removal			
· Edit Column Headers		**DatWorkflows**	
		· Affymetrix QC/Normalization (R)	
**Batch Annotations Retrieval**		· Network Inference (CLR, Pearson)	
· Gene Ontology (GO)			
· Pathways (KEGG)		Data Analysis and Visualization*	
· Gene Annotation (NCBI, CMR, miRNA)		· Clustering (MeV)	
· Protein Annotation (Uniparc, CMR)		· Network Visualization (Cytoscape)	
· Protein Interactions (BIND, Prolinks)		· Functional Enrichment (DAVID)	

### miRNA Targets

Predicted gene targets can be retrieved from the miRNA Target interface for any list of miRNAs through TargetScan [2], microCosm/miRBase [26] and miRNA.org [7] databases, which utilize TargetScanS or miRanda algorithms. Because individual prediction databases may provide false positive targets [27], we made it possible to identify high-confidence targets in BRM that are conserved across any two databases or in all three databases. Alternatively, it is also possible to retrieve all targets that appear in any database for use in downstream analysis.

### miRNA Metadata

The miRNA Metadata retrieval provides mature accession and sequence information for input miRNAs, which is useful for querying external miRNA databases or when comparing miRNA homology for different species.

### miRNA IDs

It is also possible to identify all miRNAs associated with a gene of interest or list of genes through the miRNA ID interface. This retrieval window will query genes in several formats, including gene symbol, GenBank accession, Ensembl transcript or Entrez Gene, to output miRNAs from micro/Cosm/miRBase, TargetScan and microRNA.org.

### XSpecies identifier

The cross-species identifier retrieval uses Ensembl Gene ID for species mapping making it easy to identify orthologous genes among human, mouse, rat, macaque or zebrafish. The retrieval also provides the percent identity match for both the source and destination species. This cross-species functionality expands the ability of BRM to provide batch annotations (e.g. GO or KEGG), particularly for species such as zebrafish that may have limited annotation available. Instead, human orthologs can be used for improved annotation retrieval from these databases. In addition, queries from both the original and mapped species can be merged in BRM to provide even better annotation. The mapped ortholog data can also be exported from BRM for use in other software programs that may only support a limited number of species, such as human or mouse.

### Example annotation, cross-reference and cross-species retrievals for zebrafish

One of the primary conveniences of BRM is the ability to map identifiers across databases (NCBI, UniProt, Ensembl, GO, KEGG) in the same software program used to merge and analyze datasets. It is a common problem for researchers to be limited in their ability to compare or merge data because of the lack of a common identifier, whether this is due to differences in platform (e.g. Agilent v. Affymetrix), data type (e.g. transcriptomic v. proteomic) or species. In BRM v2.3, we have expanded the number of species that can be used for retrieval and added additional retrieval options. Many queries, including those from UniProt, NCBI and GO, are available for all species, while CMR queries are available for microbial species. KEGG pathway and gene retrievals can now be made for approximately 50 of the most common eukaryotic and prokaryotic organisms. All cross-species and miRNA retrievals are currently available for human, mouse, rat, macaque and zebrafish.

Here we present example batch retrievals in zebrafish (*Danio rerio*) for annotation, cross-reference and cross-species queries (Figure 1B). By starting with only a single column of zebrafish identifiers (e.g. Entrez Gene ID) as input, we show retrieval of (1) gene symbol and Ensembl gene ID from the Gene (NCBI) Data query; (2) biological process GO term from the GO Data query; (3) pathway names from the KEGG Data query; and (4) UniProt/SwissProt accession from the Protein (UniParc) Data query. Further, by using the zebrafish Ensembl gene as input for the XSpecies Identifier query, we can map the zebrafish genes to human Ensembl Gene ID for additional data mining. We can also use either the Gene or Protein Data queries to cross-reference identifiers and retrieve Ensembl transcript ID for use in the MicroRNA ID query. All query interfaces allow the user to choose the appropriate species, input ID/column and output ID for retrieval. The queries occur in the background through internal conversion tables and the output can be added as columns to the current spreadsheet or as a separate tab. These features of BRM v2.3 allow for seamless processing of HTP data for zebrafish and many other species.

### Example workflow for integration and analysis of miRNA and mRNA microarray data

**Figure 2:**
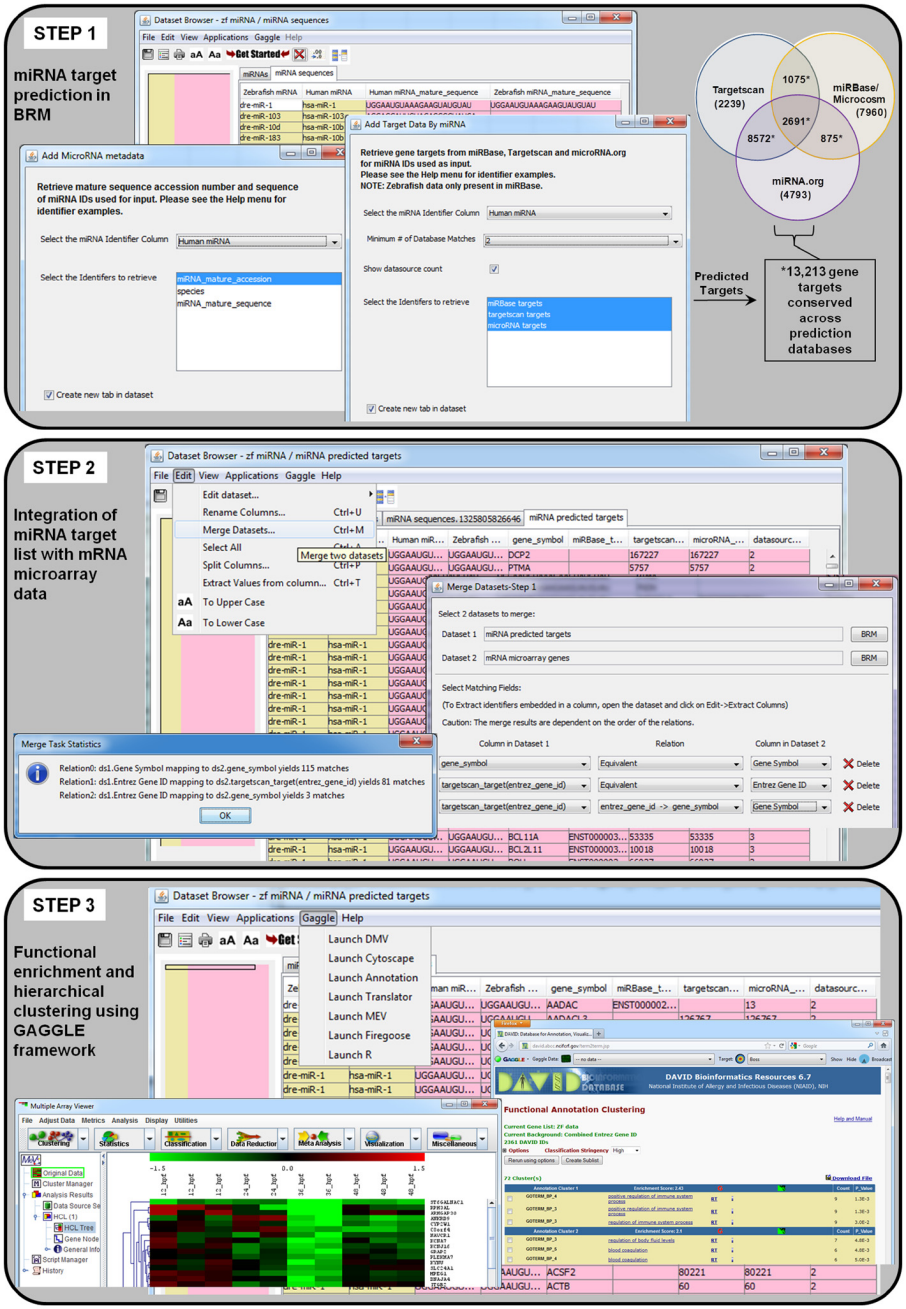
**Example workflow for integration and analysis of miRNA and mRNA microarray data.** The steps required in BRM v2.3 for (**1**) miRNA target prediction, (**2**) integration of miRNA target list with mRNA microarray data, and (**3**) functional enrichment and hierarchical clustering of the integrated miRNA target list using a GAGGLE framework.

In order to understand the biological consequences of miRNA expression changes, it is necessary to know which miRNA target genes might be post-transcriptionally repressed within a given biological system. Because target prediction algorithms identify hundreds of possible targets, accurate identification of putative mRNA targets can be illuminated from parallel experimental measurements of miRNAs and mRNAs (e.g. microarray or RNAseq) followed by computational analysis involving (1) miRNA target prediction, (2) integration of predicted targets with mRNA transcripts, and (3) functional and pathway analysis of resulting experimental miRNA gene targets. BRM v2.3 provides a convenient platform for performing these steps within a single software environment (Figure 2) instead of multiple manual steps in separate tools.

In this example, we wanted to determine whether transient developmental exposure to the neurotoxicant nicotine misregulates expression of miRNAs that control neurobehavioral development and function. Therefore, zebrafish embryos were exposed to 30 μM nicotine from 6–48 hours post fertilization (hpf), a window that encompasses early neurogenesis, and samples were collected at multiple developmental stages for parallel miRNA and mRNA microarray analysis (Additional file 1). Importantly, transient developmental exposure to nicotine resulted in behavioral hyperactivity in larval zebrafish in the absence of overt morphological defects (Additional file 2). To identify miRNA and putative target transcripts that may drive the observed behavioral phenotype, significant gene lists for each dataset were uploaded to BRM for integration and analysis of miRNA target genes.

### Step 1. miRNA target prediction

Developmental exposure of zebrafish to nicotine resulted in alteration of 42 significant (p<0.05) miRNAs across all developmental stages compared to control animals (Additional file 3). Because zebrafish miRNA target prediction databases are limited, we uploaded the zebrafish miRNA list with human homologs identified from miRBase to BRM in order to retrieve predicted targets from all three prediction datasources. The orthologous human and zebrafish miRNAs were identified based on coding sequence. In BRM, we used the miRNA Metadata query to retrieve mature sequences for both species and then filtered our list to the 33 miRNAs with ≤ 1 mismatch (but with perfect complementarity in the seed region) using the integrated OpenOffice Calc feature. These highly conserved human miRNAs were submitted to the miRNA Target query for retrieval of human predicted gene targets that were conserved across any 2 of the 3 datasources (TargetScan, microCosm/mirBase, and miRNA.org) using an approach described previously [28]. The target algorithms consider the presence of the 3’ binding site for prediction of target genes [2,7,26]. As depicted in the Venn diagram (Figure 2, Step 1), 28,205 targets with unique gene symbols were predicted from all three sources, including 14,577 from TargetScan, 12,601 from microCosm/mirBase and 16,931 from miRNA.org. A total of 13,213 targets matched at least 2 of the 3 databases and were used for integration with the mRNA transcripts in Step 2. In contrast, it is possible to directly query the zebrafish miRNAs for predicted targets in BRM using the target query from microCosm/mirBase, which is currently the only miRNA target prediction tool to support this species. This retrieval results in 4,192 zebrafish targets with unique gene symbol, which could either replace or be used in combination with the human predicted target list.

### Step 2. Integration of miRNA target and mRNA microarray gene lists

The next step is to integrate the miRNA target list with the mRNA microarray gene list (Figure 2, Step 2). Developmental exposure to zebrafish resulted in 496 genes significantly altered (p<0.05) compared to controls as measured by Nimblegen microarray. For data integration, the Merge Datasets function can be accessed from the Edit menu within the open miRNA target prediction dataset from Step 1. The mRNA transcript file is then selected for merging through a browse feature which allows selection of any file in the BRM project menu. Next, the columns to merge on must be chosen. BRM allows multi-level merges so that it is possible to achieve the best overlap between datasets through both direct relationships (i.e. common identifiers between datasets) or indirect relationships that involve translation of one identifier to another (e.g. Entrez Gene → gene symbol). BRM will merge based on the order of the relations shown, so in this case gene symbol is merged, followed by entrez IDs, finally attempting to merge any remaining identifiers by mapping the entrez ID to previously unmapped gene symbols. The user can then select which columns to show in the output, whether to show only matching rows (intersection) or all rows from both datasets (union), as well as the location to save the merged dataset. After clicking the Merge button, a pop up window shows the merge statistics of each relationship.

Overall, 199 mRNA transcripts (out of 496) matched predicted targets of altered miRNAs. These data suggest that transient developmental exposure to nicotine results in differential expression of gene transcripts putatively targeted by miRNAs significantly misregulated upon nicotine exposure. In this example, we merged all mRNA transcripts with conserved miRNA gene targets; however, it is also common to merge only anti-correlated mRNAs with miRNA targets [28] since a primary mechanism for miRNAs to direct post-transcriptional regulation of proteins is through repression. Anti-correlated lists would first need to be separated into up and down datasets and could then be merged as described above.

### Step 3. Functional enrichment and hierarchical clustering using GAGGLE framework in BRM

In Step 3, we predict the functional consequences of nicotine-mediated disruption of miRNA signaling pathways in developing zebrafish (Figure 2). Gaggle is used to broadcast data from BRM to other analysis programs, including DAVID (via Firegoose) for functional enrichment and MEV for clustering analysis. First, in order to visualize the changes associated with putative miRNA transcripts after exposure to nicotine, we clustered the data using MEV. To do this, we simply highlighted the identifier column along with all of the expression data columns in BRM to broadcast to MEV. Unsupervised hierarchical clustering then was performed in MEV by Euclidean distance metric and centroid linkage clustering to group patterns of gene expression across the timecourse.

**Figure 3:**
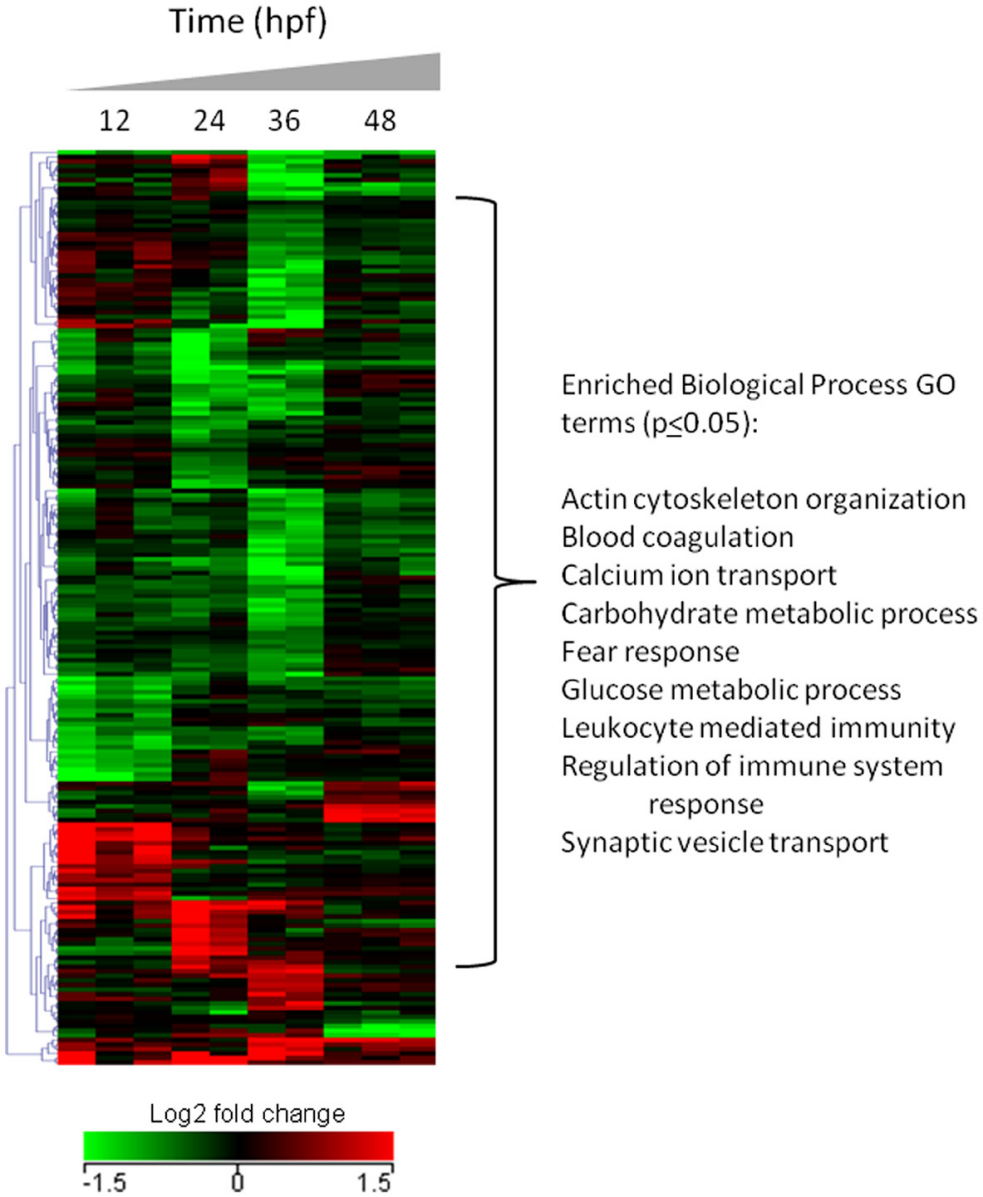
**Functional enrichment and hierarchical clustering of putative miRNA target genes using GAGGLE framework in BRM. ** Hierarchical clustering and functional enrichment of the 199 mRNA transcripts putatively regulated by miRNAs as a result of processing through the miRNA workflow in Figure 2. These miRNA gene targets were significantly regulated by 30 uM nicotine exposure in developing zebrafish from 6–48 hours post fertilization and support the role of miRNAs in regulating nervous system development and function among other important biological processes.

To identify biological processes enriched in predicted miRNA targets, we performed functional enrichment of the data in DAVID. Once the Firegoose option is chosen from the dataset browser in BRM, a Firefox window will open and connect the Firegoose toolbar to the Gaggle boss. It is then possible to broadcast a column of gene identifiers to DAVID via Firegoose. In this example, the gene symbol column from the integrated mRNA/miRNA dataset was broadcast to DAVID for functional enrichment using the Nimblegen array platform as the background. We utilized the DAVID clustering annotation tool to identify significantly enriched (p≤0.05) biological process GO terms for the 199 miRNA gene targets. Figure 3 shows the resulting functional enrichment and clustering analysis of the integrated miRNA predicted target and mRNA transcript dataset. Enrichment of biological processes related to immune function (GO:0002684, GO:0002443), blood coagulation (GO:0007596), metabolic processes (GO:0006096, GO:0006006) and cytoskeleton organization (GO:0030036) were observed. In addition, several processes related to nervous system development and function were also altered, including fear response (GO:0042596), synaptic vessicle transport (GO:0048489) and calcium ion transport (GO:0051928), indicating that nicotine exposure disrupts the expression of genes involved in neurogenesis, possibly through post-transcriptional regulation by differentially expressed miRNAs. These data identify a suite of misexpressed miRNAs and putative target transcripts that may choreographbehavioral hyperactivity in zebrafish developmentally exposed to nicotine. More broadly, the findings open the door for targeted studies to identify the mechanism by which developmental exposure to nicotine produces behavioral abnormalities in larval zebrafish. As this analysis was only performed for miRNAs that are highly conserved across species, the data may reveal insights into the role of miRNA signaling during development and the functional consequences of developmental nicotine exposure in higher vertebrate organisms. Collectively, these data support the concept that miRNA signaling pathways are targets of developmental neurotoxicants and can be altered by developmental nicotine exposure.

## Conclusions

BRM v2.3 has the capability to query predicted miRNA targets from multiple databases (TargetScan, MicroCosm/miRBase, miRNA.org), retrieve potential regulatory miRNAs for known genes, integrate experimentally derived miRNA and mRNA datasets, perform ortholog mapping across species, and retrieve annotation and cross-reference identifiers for an expanded number of species. These tools combined with the ability of BRM to directly broadcast to publically available tools for additional visualization and biological enrichment analysis provides a platform for seamless data analysis. We provide example workflows using mRNA and miRNA microarray data collected after developmental exposure of zebrafish to nicotine to successfully determine (1) the differentially expressed mRNA transcripts that are putatively regulated by misexpression of miRNAs and (2) the biological consequences of nicotine in altering miRNA signaling pathways during development. Overall, BRM v2.3 provides computational tools with a simple user interface necessary for biologists to perform miRNA data analysis and cross-species mapping as part of an integrated software environment that supports data management, mining, integration and functional annotation of HTP datasets.

## Availability and requirements

**Project name:** Bioinformatics Resource Manager (BRM) v2.3

**Project home page:**http://www.sysbio.org/dataresources/brm.stm

**Operating system(s):** Platform independent

**Programming language:** Java

**Other requirements:** Java Version 6 Update 18 or higher

**License:** Freeware

**Any restrictions to use by non-academics:** None

## Abbreviations

CMR: Comprehensive microbial resource; BRM: Bioinformatics resource manager; EDMS: Experimental data management system; GO: Gene ontology; HTP: High-throughput; ID: Identifier; KEGG: Kyoto encyclopedia of genes and genomes; miRNA: microRNA; NCBI: National center for biotechnology information; RNA: Ribonucleic acid; UniProt: Universal protein database; UTR: Untranslated region.

## Competing interests

The authors have no competing interests to declare.

## Authors’ contributions

SCT participated in the design of the software and study, performed the data analysis and drafted the manuscript. TLT and JAF carried out the molecular and biological studies and participated in the experimental design and data interpretation. SMS tested the software updates, performed data analysis and helped draft the manuscript. ESP developed the software updates and helped to draft the manuscript. RLT and KMW conceived of the study, participated in its design and coordination and helped to draft the manuscript. All authors read and approved the final manuscript.

## Supplementary Material

Additional file 1 Zebrafish nicotine exposure and neurobehavorial methods. Click here for file

Additional file 2 Abnormal neurobehavioral development in zebrafish larvae developmentally exposed to nicotine. Click here for file

Additional file 3 Zebrafish miRNAs significantly (p<0.05) regulated by transient developmental exposure to 30 μM nicotine and human orthologs. Click here for file
