# Supplementary material for: Bioinformatics resource manager v2.3: an integrated software environment for systems biology with microRNA and cross-species analysis tools
Source: BMC Bioinformatics. 2012 Nov 23;13:311. doi: 10.1186/1471-2105-13-311 (PMC3534564; doi:10.1186/1471-2105-13-311)
Supplement: Additional file 1 — Zebrafish nicotine exposure and neurobehavorial methods. [file 1471-2105-13-311-S1.pdf]

Additional File 1. Zebrafish nicotine exposure and neurobehavorial methods.

Fish care and husbandry: Adult Tropical 5D strain zebrafish (*Danio rerio*) were raised according to Institutional Animal Care and Use Committee protocols in the Sinnhuber Aquatic Research Laboratory at Oregon State University. Adults were maintained on a 14 h light/10 h dark schedule on a recirculating system in which water was maintained at 28±1 °C with a pH of 7.0±0.2.

Nicotine exposure: For phenotypic studies, embryos were waterborne exposed to 0-30 μM nicotine (Sigma) in buffered embryo medium (Westerfield, 2000) or embryo medium alone in 96 well plates beginning at 4-6 hours post fertilization (hpf). At 120 hpf, larvae were visually assessed for malformations and normal touch response and subjected to an automated behavior test. For miRNA and cDNA array studies, groups of embryos were exposed to 0 or 30 μM nicotine from 4-48 hpf in 20-ml glass vials sealed with Teflon-lined lids. Each exposure group was considered a single replicate. At 48 hpf, embryo homogenate was collected. RNA isolation, miRNA isolation, cDNA synthesis, labeling, and hybridization were performed as previously reported (Tal et al., 2012).

Larval locomotion assay: At 5 dpf, automated quantification of larval movement was obtained during alternating 5 minute periods of light and dark (MacPhail et al., 2009). For all experiments, larvae were subjected to locomotion analyses at 12:00-15:00 hr in a sound and temperature controlled (26°C) behavioral testing room. Movement of 96 individual larvae were simultaneously tracked and recorded using an automated video tracking system (Videotrack, quantization mode; Viewpoint Life Sciences, Lyon, France). To distinguish 5 dpf larvae within each individual well from the background, the life stage-specific size parameter was set at 17 and the number of seconds during which the larvae moved above the movement threshold, set at 7, was analyzed in 60 s bins. The data was processed using a custom PERL script. Data are presented as the total time spent in motion (sec) during the 5 min dark period. Data were

analyzed using a repeated measures analysis of variance (ANOVA) with a Tukey's Multiple Comparison Post Hoc Test (n=16 animals per group).

References:

MacPhail, R. C., Brooks, J., Hunter, D. L., Padnos, B., Irons, T. D., and Padilla, S. (2009) Locomotion in larval zebrafish: Influence of time of day, lighting and ethanol. *Neurotoxicology* **30**, 52-58

Tal, T. L., J. A. Franzosa, et al. (2012). "MicroRNAs control neurobehavioral development and function in zebrafish." FASEB J **26**(4): 1452-1461.

Westerfield, M. (2000) The zebrafish book. 4th ed. *Univ. of Oregon Press, Eugene.*
