# Supplementary material for: Bioinformatics resource manager v2.3: an integrated software environment for systems biology with microRNA and cross-species analysis tools
Source: BMC Bioinformatics. 2012 Nov 23;13:311. doi: 10.1186/1471-2105-13-311 (PMC3534564; doi:10.1186/1471-2105-13-311)

**Additional File 2. Abnormal neurobehavioral development in larvae developmentally exposed to nicotine. (A-C)** Embryos were individually exposed to nicotine (0-30 μM) in a 96 well plate from 4-120 hpf. At 120 hpf, larvae were visually assessed for malformations and subjected to a touch response test or automated locomotion test. **(A)** Nicotine exposure does produce in overt malformations. **(B)** % Abnormal touch behavior at 120 hpf. **(C)** Nicotine exposure results in concentration-dependent behavioral hyperactivity during the dark period. Significance was determined by one-way ANOVA with a Tukey's post test (** p<0.01, *** p<0.001).

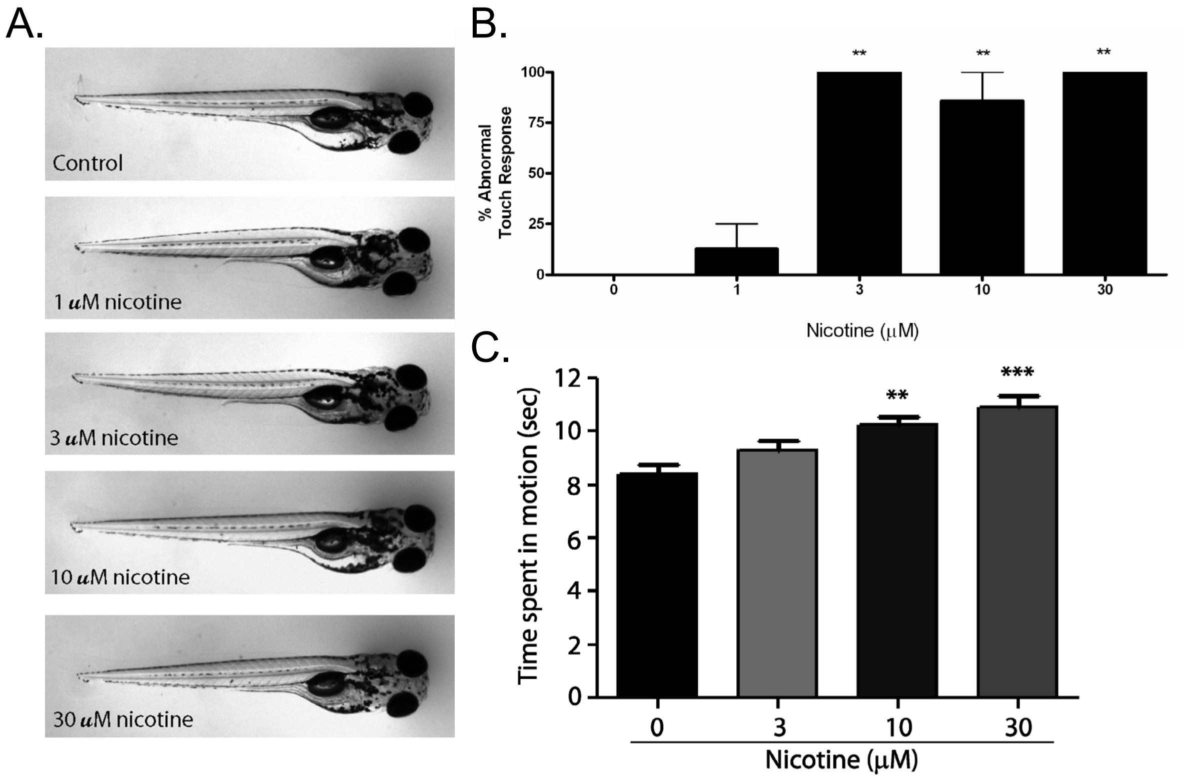

Supplement: Additional file 2 — Abnormal neurobehavioral development in zebrafish larvae developmentally exposed to nicotine. [file 1471-2105-13-311-S2.pdf]
