# Supplementary material for: Bioinformatics resource manager v2.3: an integrated software environment for systems biology with microRNA and cross-species analysis tools
Source: BMC Bioinformatics. 2012 Nov 23;13:311. doi: 10.1186/1471-2105-13-311 (PMC3534564; doi:10.1186/1471-2105-13-311)
Supplement: Additional file 3 — Zebrafish miRNAs significantly (p<0.05) regulated by transient developmental exposure to 30 μM nicotine and human orthologs. [file 1471-2105-13-311-S3.pdf]

Additional File 3. Human and zebrafish miRNA orthologs

Zebrafish miRNA[1]	Human miRNA[2]	Zebrafish Mature Sequence[3]	Human Mature Sequence[3]
dre-miR-1	hsa-miR-1	uggaauguaaagaaguauguau	uggaauguaaagaaguauguau
dre-miR-103	hsa-miR-103	agcagcauuguacagggcuauga	agcagcauuguacagggcuauga
dre-miR-10b	hsa-miR-183	uauggcacugguagaauucacuG	uauggcacugguagaauucacu
dre-miR-10d	hsa-miR-10b	uacccuguagaaccgaauGugug	uacccuguagaaccgaauuugug
dre-miR-128	hsa-miR-128	ucacagugaaccggucucuuuU	ucacagugaaccggucucuuu
dre-miR-133a	hsa-miR-133a	uuuggucccccuucaaccagcug	uuuggucccccuucaaccagcug
dre-miR-138	hsa-miR-138	agcugguguugugaaucaggcc-	agcugguguugugaaucaggccg
dre-miR-152	hsa-miR-152	ucagugcaugacagaacuuUgg	ucagugcaugacagaacuugg
dre-miR-153c	hsa-miR-153	uugcauagucacaaaaAugauc	uugcauagucacaaaagugauc
dre-miR-155	hsa-miR-155	uuaaugcuaaucgugauaggggg-	uuaaugcuaaucgugauaggggu
dre-miR-16b	hsa-miR-16	uagcagcacguaaauauuggAg	uagcagcacguaaauauuggcg
dre-miR-17a	hsa-miR-17	caaagugcuuacagugcaggua-	caaagugcuuacagugcagguag
dre-miR-181a	hsa-miR-181a	aacauucaacgcugucggugagu	aacauucaacgcugucggugagu
dre-miR-181b	hsa-miR-181b	aacauucauugcugucggugggg-	aacauucauugcugucgguggggu
dre-miR-183	hsa-miR-10b	uacccuguagaaccgaauuugug	uacccuguagaaccgaauuugug
dre-miR-18a	hsa-miR-18a	uaaggugcaucuagugcagaua-	uaaggugcaucuagugcagauag
dre-miR-19a	hsa-miR-19a	ugugcaaaucuaugcaaaacuga	ugugcaaaucuaugcaaaacuga
dre-miR-205	hsa-miR-205	uccuucauuccaccggagucug	uccuucauuccaccggagucug
dre-miR-206	hsa-miR-206	uggaauguaaggaagugugugg	uggaauguaaggaagugugugg
dre-miR-20a	hsa-miR-20a	uaaagugcuuauagugcagguag	uaaagugcuuauagugcagguag
dre-miR-20b	hsa-miR-20b	caaagugcucaCagugcagguag	caaagugcucauagugcagguag
dre-miR-21	hsa-miR-21	uagcuuaucagacugGuguuggc	uagcuuaucagacugauguuga
dre-miR-210	hsa-miR-210	cugugcgugugacagcggcuAa	cugugcgugugacagcggcuga
dre-miR-216a	hsa-miR-216a	uaaucucagcuggcaacuguga	uaaucucagcuggcaacuguga
dre-miR-216b	hsa-miR-216b	uaaucucugcaggcaaCuguga	aaaucucugcaggcaaauguga
dre-miR-22a	hsa-miR-22	aagcugccagCugaagaacugu	aagcugccaguugaagaacugu
dre-miR-23b	hsa-miR-23b	aucacauugccagggauuaccA	aucacauugccagggauuacc
dre-miR-24	hsa-miR-24	uggcucaguucagcaggaacag	uggcucaguucagcaggaacag
dre-miR-30a	hsa-miR-30d	uguaaacauUcccgacuggaag	uguaaacauccccgacuggaag
dre-miR-34	hsa-miR-34a	uggcagugucuuagcugguugu	uggcagugucuuagcugguugu
dre-miR-454b	hsa-miR-454	uagugcaauauugcuuauaggg-	uagugcaauauugcuuauagggu
dre-miR-499	hsa-miR-499-5p	uuaagacuugcagugauguuuA	uuaagacuugcagugauguuu
dre-miR-9	hsa-miR-9	ucuuugguuaucuagcuguauga	ucuuugguuaucuagcuguauga

[1]Zebrafish miRNAs significantly ($p<0.05$) regulated by transient developmental exposure to 30 μM nicotine as compared to control embryos. Only miRNAs that are highly conserved with human based on mature sequence ($\leq$1 mismatch and perfect complementarity in the seed region) are listed.
[2]Human homologs of zebrafish miRNAs as determined in miRBase.
[3]miRNA mature sequence retrieved from the miRNA Metadata query in BRM. Single mismatches between zebrafish and human are shown using capitalized nucleotide or dash in zebrafish sequence.
